# Clonal Patch Size and Ramet Position of *Leymus chinensis* Affected Reproductive Allocation

**DOI:** 10.1371/journal.pone.0140350

**Published:** 2015-10-15

**Authors:** Chan Zhou, Zhengwen Wang, Junyue Guo, Zhuo Zhang, Yunfei Yang

**Affiliations:** 1 School of Life Sciences, Liaoning University, No 66 Chongshan Road, Shenyang, 110036, People’s Republic of China; 2 Key Laboratory for Vegetation Ecology, Ministry of Education, Institute of Grassland Science, Northeast Normal University, No 5268 Renmin Street, Changchun, 130024, People’s Republic of China; 3 State Key Laboratory of Forest and Soil Ecology, Institute of Applied Ecology, Chinese Academy of Sciences, Shenyang, 110164, People’s Republic of China; 4 College of Biology Science and Bioengineering, Shenyang University, No 21 Wanghua Street, Shenyang, 110044, People’s Republic of China; Swiss Federal Research Institute WSL, SWITZERLAND

## Abstract

Reproductive allocation is critically important for population maintenance and usually varies with not only environmental factors but also biotic ones. As a typical rhizome clonal plant in China's northern grasslands, *Leymus chinensis* usually dominates the steppe communities and grows in clonal patches. In order to clarify the sexual reproductive allocation of *L*. *chinensis* in the process of the growth and expansion, we selected *L*. *chinensis* clonal patches of a range of sizes to examine the reproductive allocation and allometric growth of the plants. Moreover, the effects of position of *L*. *chinensis* ramets within the patch on their reproductive allocation were also examined. Clonal patch size and position both significantly affected spike biomass, reproductive tiller biomass and SPIKE/TILLER biomass ratio. From the central to the marginal zone, both the spike biomass and reproductive tiller biomass displayed an increasing trend in all the five patch size categories except for reproductive tiller biomass in 15–40m^2^ category. *L*. *chinensis* had significantly larger SPIKE/TILLER biomass ratio in marginal zone than in central zone of clonal patches that are larger than 15 m^2^ in area. Regression analysis showed that the spike biomass and SPIKE/TILLER biomass ratio were negatively correlated with clonal patch size while patch size showed significantly positive effect on SEED/SPIKE biomass ratio, but the reproductive tiller biomass and SEED/TILLER biomass ratio were not dependent on clonal patch size. The relationships between biomass of spike and reproductive tiller, between mature seed biomass and spike biomass and between mature seed biomass and reproductive tiller biomass were significant allometric for all or some of patch size categories, respectively. The slopes of all these allometric relationships were significantly different from 1. The allometric growth of *L*. *chinensis* is patch size-dependent. This finding will be helpful for developing appropriate practices for the management of *L*. *chinensis*-dominant grasslands.

## Introduction

A more accurate estimate of the relative contribution of each individual to the next generation is reproductive rather than vegetative in clonal plant species [1]. Variability in reproductive allocation under different environments is essential for the processes of adaption and evolution [2]. Reproductive allocation refers to the proportion of total biomass allocated to reproductive tissues [3, 4], and thus reflects the balance between reproductive and vegetative growth. The responses of reproductive allocation to abiotic environmental changes and/or anthropogenic disturbances are a core part of plant life history theory [5–7]. In changing environments, plants modify their resource allocation to adapt to the environmental constraints [8–14]. For instance, plant reproductive allocation might be reduced by water stress [2], but be enhanced by salinity [15].

Reproductive allocation can also be affected by biotic factors, such as competition [1, 16–18], plant density [19] and clonal patch size [20]. For example, allocation to sexual reproduction of *Geum reptans* could be reduced when growing with *Poa*. *alpina* [21]. Biomass allocation to sexual reproduction of *Arum italicum* increases with increasing clonal plant size, leading to the depression of allocation to vegetative reproduction [22]. *Trifolium* stolons grew longer and faster at the border than at the inside on clonal patches [20]. Therefore, we assume that reproductive allocation of *Leymus chinensis* ramets would be affected not only by clonal patch size, but also by the position of the ramets within a clonal patch. This might be the result that ramets in different positions within the patch are exposed to different ramet densities, and therefore differ in inter-ramet competition. However, it is still unclear whether and how sexual reproductive allocation is affected by clonal patch size and the location of clonal ramets within a clonal patch.

Plant growth is widely believed to be allometric [7, 23–27]. Allometric growth is defined as the relative growth of a part of an organism relative to the whole growth of that organism [23]. Reproductive allocation of plants is the most extensively studied allometric process, and is commonly believed to be dependent on plant size [19, 28–31]. Recently, the allometry theory was used to disentangle the size-dependent effect from the variation of plant reproductive allocation [32–34]. The equation y = βx^α^, or more commonly Log(y) = Log (β) + αLog (x), is generally used to describe the allometric relationships between vegetative (x) and reproductive (y) biomasses. If the value of α (i.e. slope) equals 1 or is not significantly different from 1, y is isometric with size (x); if the value of α is significantly different from 1, y is anisometric with size (x). Constant α value suggests fixed allometric relationships among treatments, and size-dependent effects play a major role in plant reproductive allocation; varying α values suggest plastic allometric relationships, and size-independent effects also play such an important role as those which are size-dependent on reproductive allocation [7, 12, 31].

Various authors have described the structure of a plant community as a spatial and temporal mosaic of patches [35–37]. Many species of flowering plants have a patchy distribution, often with large variation in patch size, especially in arid and semiarid grazing systems throughout the world [36, 37]. Knowledge of ecological processes in these vegetation patches is essential since these systems cover nearly 30% of the earth´s land surface [38]. The spatial distribution of plant populations can affect, for example, different aspects of insect-plant interactions [39] such as the behavior of flower-visiting insects [40, 41]. Thereafter, the pollination rate of plants might differ among patches of different size, with subsequent effects on sexual reproduction. Jennersten and Nilsson [42] examined if the frequency of pollinator visits on seed set changed with patch size in the non-clonal, perennial, caryophyllaceous herb *Viscaria vulgaris*. These authors reported that despite similar pollinator visitation rates and similar potentials for seed set in the various study patch sizes, natural seed set increased with patch size; they concluded that pollinators may have deposited more *Viscaria* pollen per flower visit in large than in small patches. Thus, and in spite of some work has been done on the relationship between sexual reproduction and patch size, it is limited to plants which only reproduce sexually. As a result, studies which address such a relationship on clonal plant species are critical.

As, *L*. *chinensis* is a dominant, widely distributed, typical clonal plant species in the Eurasian steppe zone [43, 44]. It has a strong capacity of vegetative reproduction [45] but low seed setting rate [46]. This is because this species propagates mainly by clonal growth[47, 48]. Consequently, many *L*. *chinensis* ramets can be propagated from a single original ramet and form a clone; therefore, a given set of clones would ultimately appear as clonal patches of different sizes. It remains unclear whether and how the sexual reproductive allocation changes during the expansion of *L*. *chinensis* clones. Moreover, it has not been reported whether the position that a ramet occupies within a clonal patch affects reproduction allocation. We examined reproductive allocation patterns of *L*. *chinensis* in patches of different sizes and analyzed allometric relationships to answer these questions. Specifically, we addressed the hypotheses that: (1) reproductive allocation of a ramet growing in a clone increases from the periphery to the center of the clonal patch, (2) clonal ramets increase their sexual reproductive allocation with the increase of clonal patch size, and (3) the allocation to sexual reproduction of *L*. *chinensis* ramets is allometric during the process of clonal expansion.

## Materials and Methods

### Site description

This study was conducted in the Grassland Ecology Field Station of Northeast Normal University, Changling county, Jilin province, China (123°31' E, 44°45' N), located at the south of the Songnen Plain, northeast China. The grassland is flat and low-lying, with only slight topographical undulations, and is characterized by a warm temperate, semi-humid to semi-arid climate. Annual precipitation is usually 350–500mm, 70% of which falls from June to September. The frost-free period is 136–163 days. The soil type of the plain corresponds to a salinized meadow soil. Vegetation is dominated by *L*. *chinensis*, accompanied by *Phragmites communis* and *Carex duriuscula* among other plant species.

### Species

*Leymus chinensis* is a typical perennial rhizome clonal plant, which grows well on many soil types, such as dark brown, saline meadow, chernozem and chestnut soils [49]. It has a high forage value because of its high palatability and nutritional value [50]. The vegetative reproduction of *L*. *chinensis*, a highly adapted species in the study region [51], is vigorous: it has long, strong rhizomes which give rise to extensively spreading clones. The rhizomes lie horizontally to the soil surface at about 20cm soil depth; their diameters are usually 2–3 mm with internode lengths of 2–6 cm [45]. However, the capacity of sexual reproduction of *L*. *chinensis* is rather weak under natural conditions. This is reflected on its low earing, seed setting and germination rates [52]. It flowers in early June and ripens in mid-July [53].

### Experimental design

**Fig 1:**
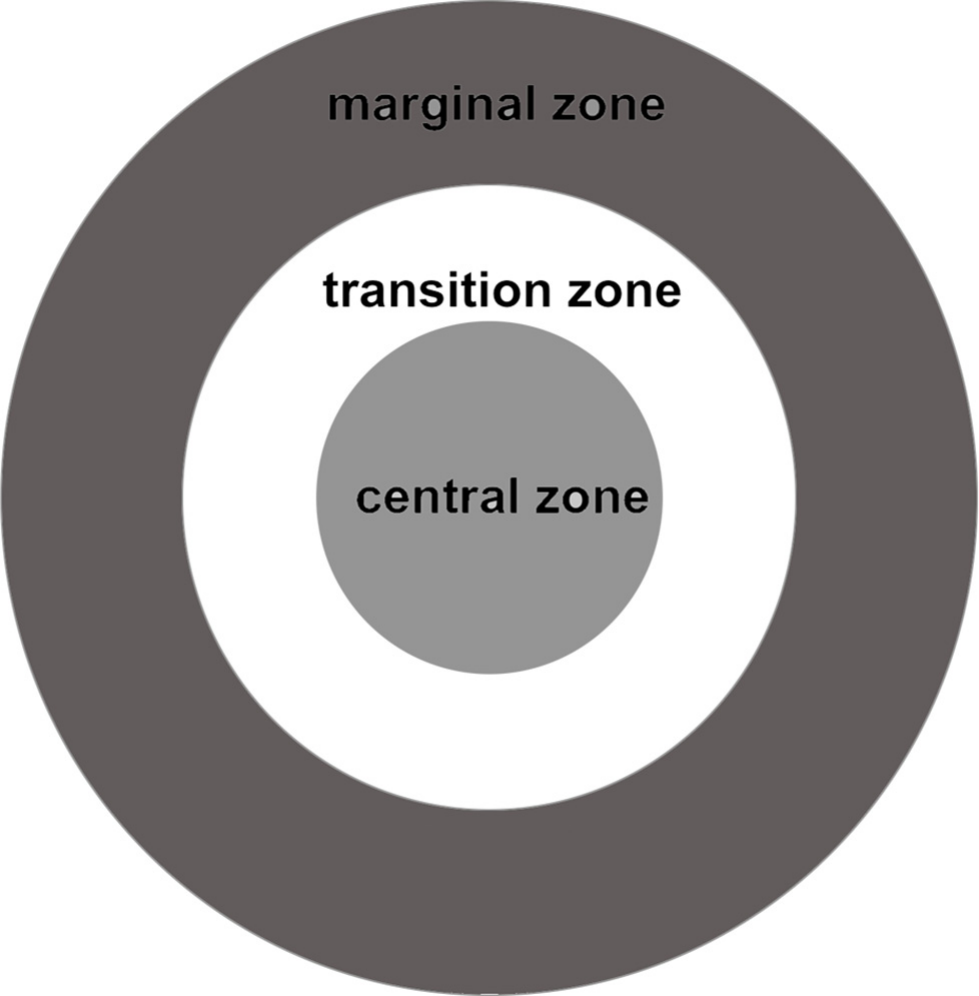
The central, transition and marginal zones of a *Leymus chinensis* clonal patch are indicated.

A total of 14 clonal patches of *L*. *chinensis*, ranging from 2.78m^2^ to 166.79m^2^ in patch size were selected and sorted into five patch size categories: ≤ 5m^2^, 5–15m^2^, 15–40m^2^, 40–100m^2^ and ≥ 100m^2^. Each patch size category consisted from 1 to 4 patches. We recognize that ≥100m^2^ patch category which consisted of only 1 patch was not replicated patch because of time and budget constraints. However, reproductive allocation of *L*. *chinensis* ramet was studied at the level of ramet in this research. A lot of replicated ramets in the only ≥100m^2^ patch category were determined, which can achieve this research purpose [54]. The number of genotypes within the five patch size categories were 1, 2, 4, 5 and 9, respectively (unpublished data). In each patch, reproductive tillers were sampled at a distance of 0.5, 1, 2, 4, 8 and 16 m from the center of the patch. To avoid the effect of the sampling direction, we did exactly the same procedures on eastern, western, southern, northern, northeastern, northwestern, southeastern and southwestern sampling directions with respect to the patch center for each clonal patch. As a result, a total of 37, 196, 250, 364 and 108 tillers were sampled on each of the five patch size categories, respectively. According to the distance from the center, each patch was divided into 3 regions including a central zone, a transition zone and a marginal zone (Fig 1). The only exception was the ≤ 5m^2^ patch size category, where the sampling was conducted only at the distance of 0.5 and 1m since a transition zone was not available. The areas for the central, transition and marginal zones were approximate in every patch size category.

### Field sampling and measurement

Field sampling was conducted in July 2010, when the seeds of *L*. *chinensis* ripened. Reproductive tillers were harvested at the ground surface. Each tiller was placed into an envelope, and its position and direction within the patch were recorded. All samples were marked carefully, taken to the laboratory and dried at 65°C. For each tiller, the number of mature seeds and flowers were counted. Dry biomass was thereafter obtained for the mature seeds, spikes, and reproductive tillers. Then the following biomass rations were calculated for spike / reproductive tiller (SPIKE / TILLER), mature seed / spike (SEED / SPIKE), and mature seed / reproductive tiller (SEED / TILLER). Seed setting rate was calculated as the ratio between the numbers of seeds to that of flowers. Data is showed in S1 File.

### Data analysis

The differences in biomass allocation parameters among central, transition and marginal zones were analyzed by multiple comparison tests. Two-way ANOVA was used to examine the effects of clonal patch size and position within the patch on reproductive characteristics. For the ≤ 5m^2^ patch size category, transition zone is not available, and its corresponding data were treated as missing values. The relationships between reproductive characteristics and patch area were analyzed with regression analysis, and the best fit model was selected. These data analyses were performed with SPSS 17.0 statistics software. The model Y = βX^α^ was used to analyze the reproductive allometric growth of a tiller. The equation was log transformed as follows: log_10_{Y} = log_10_{β} + α log_10_{X}; where X was biomass of a reproductive tiller, spike biomass or reproductive tiller biomass, and Y was spike biomass, biomass of mature seeds or mature seed biomass, α was the scaling slope and β was the allometric intercept. Slopes and intercepts were determined with Standardized Major Axis (SMA) regression using the SMATR software. Firstly, the slopes were tested to to determine whether they differedfrom 1. The difference in allometric slopes among treatments was tested [7].

## Results

### Spike biomass, reproductive tiller biomass and seed setting rate

**Fig 2:**
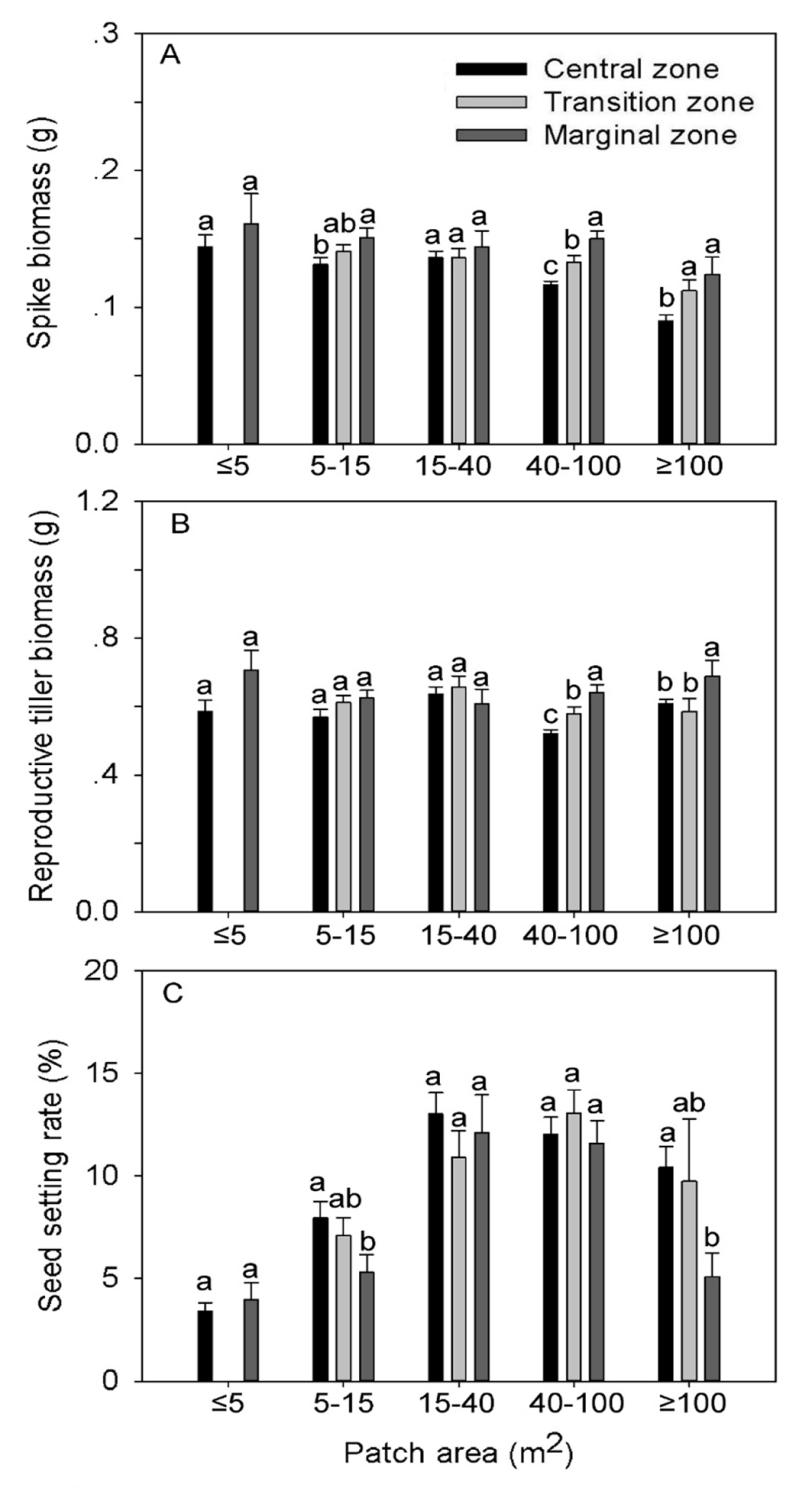
The seed setting rate, Spike (A) and reproductive tiller (B) biomasses, and seed setting rate (C) at five patch size categories of *Leymus chinensis* clones. Each histogram is the mean ± SE. Within each patch size category, different zones followed by different letters indicate significant differences at P = 0.05.

**Fig 3:**
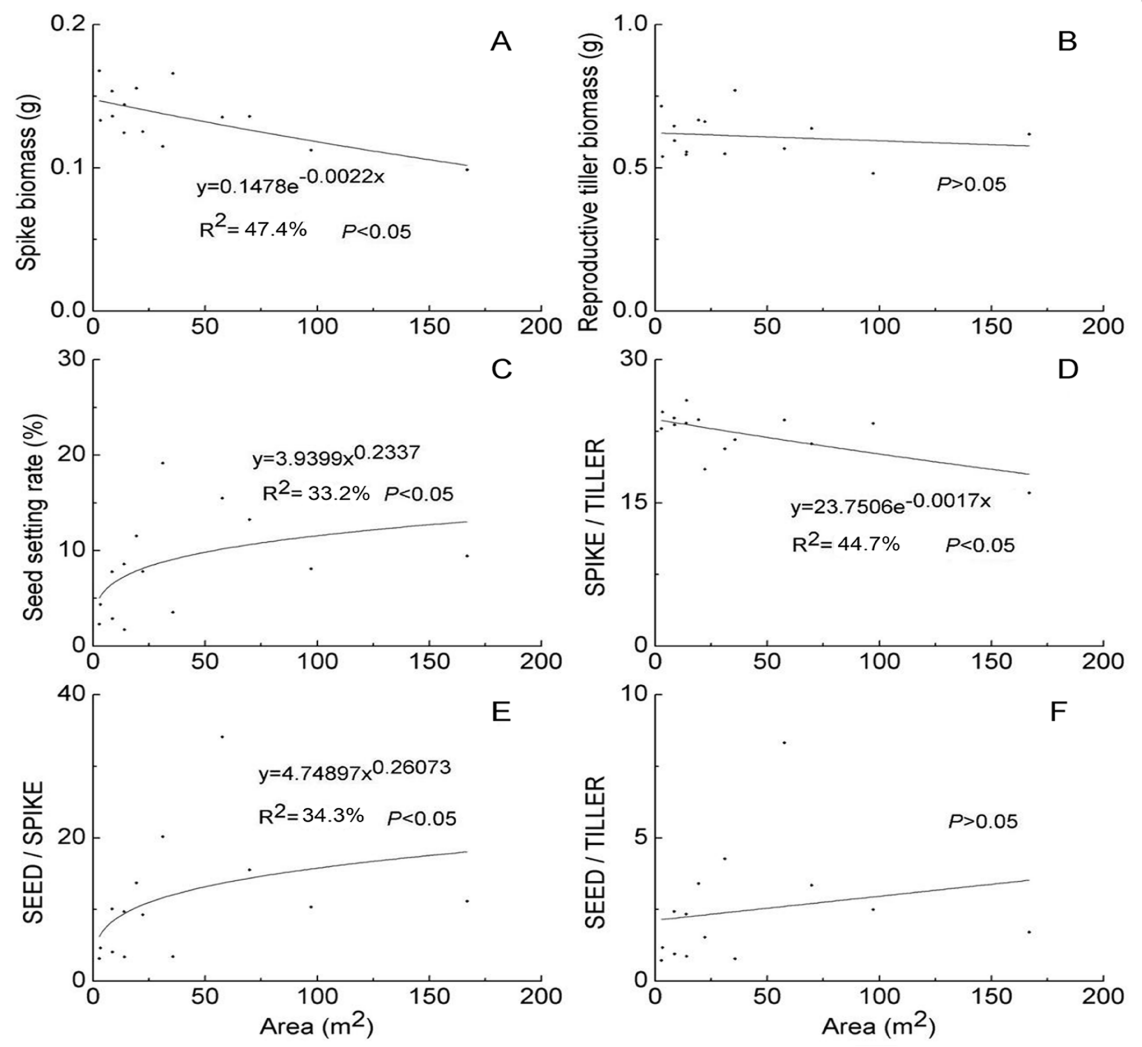
Regressions of spike biomass (A), reproductive tiller biomass (B), seed setting rate (C), spike / reproductive tiller biomass ratio (SPIKE / TILLER; D), mature seed / spike biomass ratio (SEED / SPIKE; E) and mature seed / reproductive tiller biomass ratio (SEED / TILLER; F) versus the patch area of *Leymus chinensis* clones.

Both clonal patch size and position both significantly affected spike and reproductive tiller biomass (Table 1). Meanwhile, clonal patch size but not position had significant effect on seed setting rate (Table 1). From the central to the marginal zone, both spike and reproductive tiller biomasses displayed an increasing trend in all five patch size categories (except for reproductive tiller biomass in 15–40m^2^ category). However, significant differences were only found consistently in the 40–100m^2^ and ≥ 100m^2^ patch size categories (Fig 2). By contrast, seed setting rate decreased with increasing distance from the center of patches for the 5–15m^2^ and ≥ 100m^2^ patch size categories (Fig 2). Regression analysis showed that the spike biomass was negatively correlated with clonal patch size but the reproductive tiller biomass was not dependent on clonal patch size (Fig 3). The seed setting rate increased as patch size also increased (Fig 3). The interactive effect of clonal patch size and position on seed setting rate and biomass of spike and reproductive tillers was not significant (Table 1).

**Table 1:** Effects of clonal patch size (PS) and position within the patch (patch zone, PZ) on spike and reproductive tiller biomass and seed setting rate, and the reproductive allocation parameters of *Leymus chinensis*.

Variation	df	Spike biomass			Reproductive tiller biomass			Seed setting rate		
		MS	F-ratio	*P*-value	MS	F-ratio	*P*-value	MS	F-ratio	*P*-value
PS	4	0.018	6.762	<0.001	0.109	2.543	0.038	1349.012	12.882	<0.001
PZ	2	0.027	10.153	<0.001	0.210	4.901	0.008	220.240	2.103	0.123
PS×PZ	7	0.003	1.061	0.387	0.068	1.593	0.134	85.294	0.815	0.575
Variation	df	SPIKE / TILLER			SEED / TILLER			SEED / TILLER		
		MS	F-ratio	*P*-value	MS	F-ratio	*P*-value	MS	F-ratio	*P*-value
PS	4	561.824	30.384	<0.001	5422.973	24.597	<0.001	337.388	24.499	<0.001
PZ	2	81.517	4.409	0.012	160.162	0.726	0.484	2.268	0.165	0.848
PS×PZ	7	55.480	3.000	0.004	331.912	1.505	0.162	24.285	1.763	0.091

Note: PS, Patch size; PZ, Patch zone; df, degree of freedom; MS = mean squares.

### Reproductive allocation

**Fig 4:**
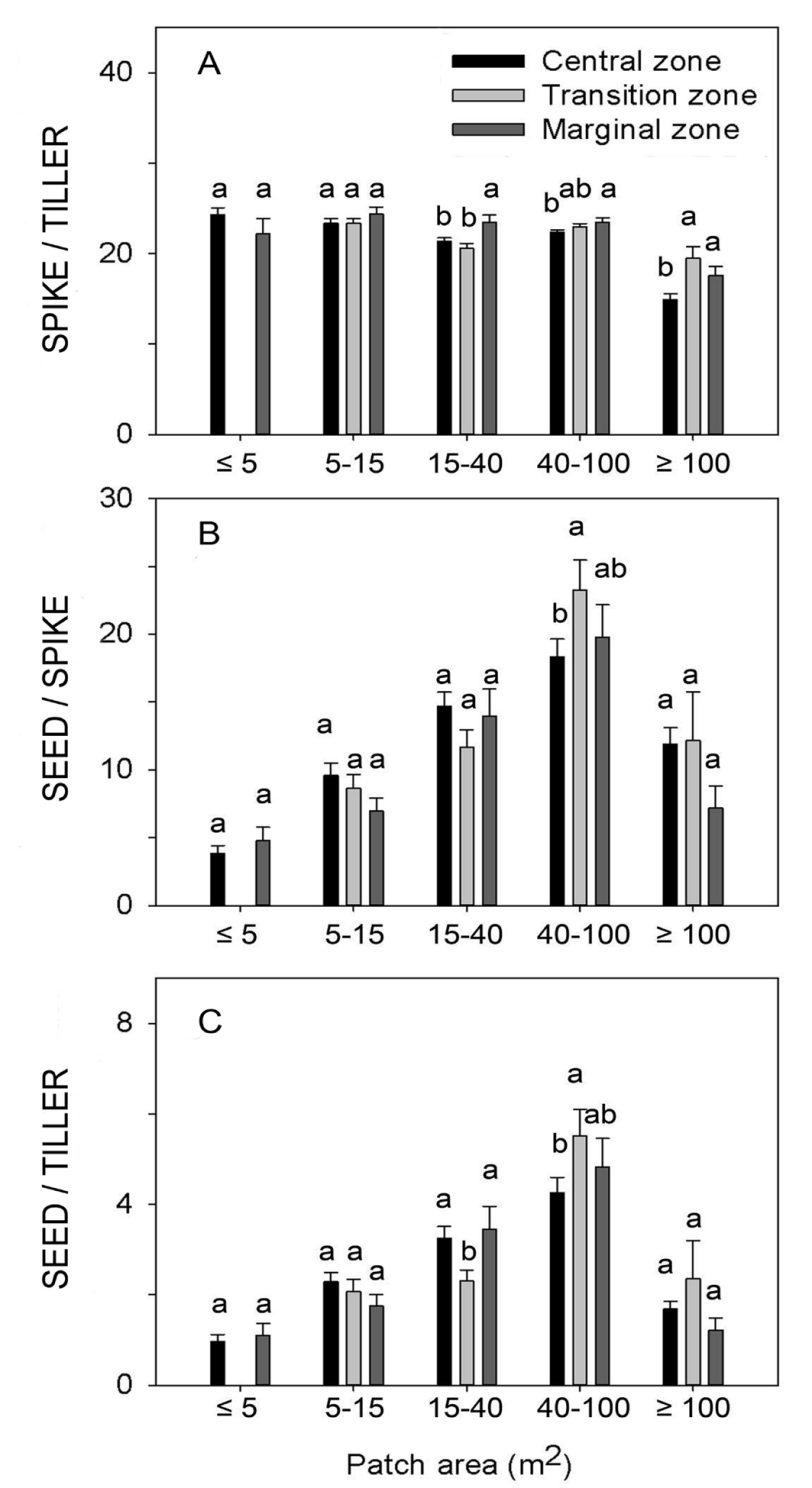
The reproductive allocation expressed as spike biomass / reproductive tiller biomass ratio (SPIKE/TILLER; A), mature seed biomass / spike biomass ratio (SEED / SPIKE; B) and mature seed biomass / reproductive tiller biomass ratio (SEED / TILLER; C) of *Leymus chinensis* clones at five patch size categories. Different zones of the same patch size category followed by the same letter did not differ significantly at P = 0.05.

Both clonal patch size and position both affected SPIKE/TILLER biomass ratio (Table 1). *L*. *chinensis* had significantly larger SPIKE/TILLER biomass ratio in marginal than in central zones of clonal patches that were larger than 15 m^2^ (Fig 4). There was a significantly negative, non-linear relationship between SPIKE/TILLER biomass ratio and patch size of *L*. *chinensis* clones (Fig 3). Meanwhile, patch size showed a significantly positive effect on SEED/SPIKE biomass ratio (Table 1, Fig 4), but the effect on SEED/TILLER biomass ratio was not significant (Fig 3). No difference was found in SEED/SPIKE biomass ratio among the three study zones on any patch size category, except for the 40–100m^2^ patch size category where the central zone showed smaller SEED/SPIKE biomass ratio than the transition one (Fig 4). For SEED/TILLER biomass ratio, no overall pattern was found among the three zones in all patch size categories (Fig 4). The interactive effect of clonal patch size and position was significant on SPIKE/TILLER biomass ratio, but not on SEED/SPIKE or SEED/TILLER (Table 1).

### Allometric relationships of reproductive allocation

**Fig 5:**
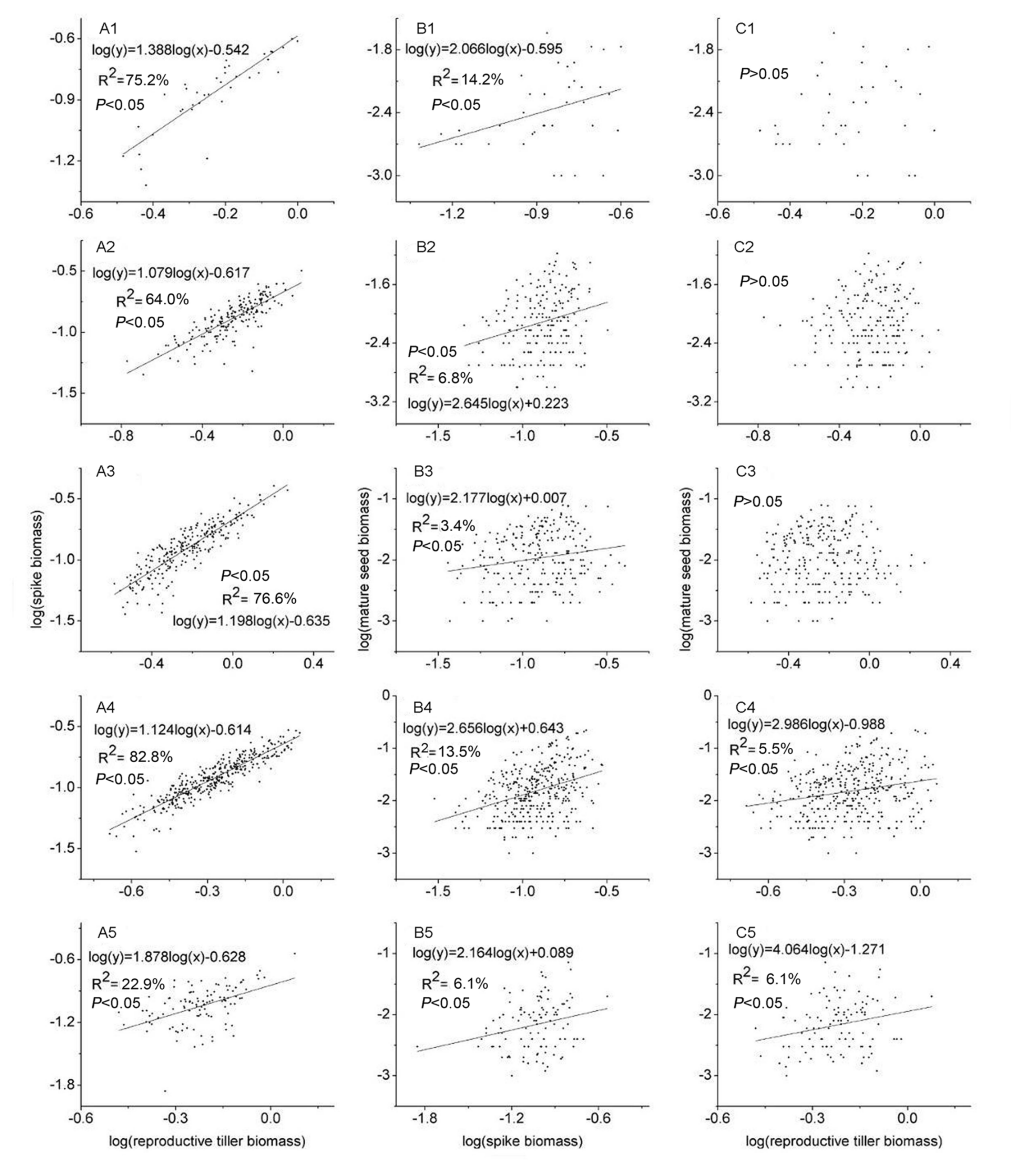
Allometric relationships of spike biomass vs. reproductive tiller biomass at the tiller level (A1-A5), mature seed biomass vs. spike biomass at the spike level (B1-B5) and mature seed biomass vs. reproductive tiller biomass at the tiller level (C1-C5) for patches with area of <5, 5–15, 15–40, 40–100 and >100m^2^, respectively. The subscript 1, 2, 3, 4 and 5 refers to clonal patches with area of <5, 5–15, 15–40, 40–100 and >100m^2^, respectively. The presence of regression lines indicates significant allometric relationship at P = 0.05.

Regression SMA analyses showed that a significant allometric relationship was found between spike and reproductive tiller biomasses for all patch size categories (Fig 5). The slopes of all these allometric relationships were significantly different from 1, except for the 5–15 m^2^ patch size category. Among these, the slopes for the ≥ 100m^2^ patch size category were significantly different from those of any other patch size category (Table 2). The relationships between mature seed biomass and spike biomass also followed significant allometric relationships (Fig 5); all the slopes were significantly different from 1 for all patche size categories. Further, the allometric slopes of the 5–15m^2^ and 40–100m^2^ patches were significantly larger than those of other three patch size categories (Table 2). For the patches of 40–100m^2^ and ≥100m^2^ size, significant allometric relationships were found between mature seed and reproductive tiller biomasses (Fig 5); the slopes of both patch size categories were significantly different from 1, and also from each other. However, this was not the case for other three patch size categories of smaller size (Table 2).

**Table 2:** Effects of clonal patch area of *Leymus chinensis* on the slopes and intercepts of reproductive allometric relationships at two levels.

log_10_Y	Area (m^2^)				
vs.log_10_X	≤ 5	5–15	15–40	40–100	≥ 100
Spike biomass vs. reproductive tiller biomass					
Slope	**1.388**b	1.079d	**1.198**bc	**1.124**cd	**1.878**a
Intercept	-0.5415	-0.6167	-0.6354	-0.6139	-0.6284
R^2^	0.752	0.640	0.766	0.828	0.229
Mature seed biomass vs. spike biomass					
Slope	**2.066**c	**2.645**ab	**2.177c**	**2.656**a	**2.164**bc
Intercept	-0.5945	0.2231	0.0066	0.6425	0.0885
R^2^	0.142	0.068	0.034	0.135	0.061
Mature seed biomass vs. reproductive tiller biomass					
Slope	-	-	-	**2.986b**	**4.064**a
Intercept	-	-	-	-0.9883	-1.2714
R^2^	-	-	-	0.055	0.061

Note: Using regression SMA analyses, allometric slopes and intercepts were obtained from the log–log linear relationship described as log_10_Y = Slope×log_10_X+Intercept. R^2^ values indicate the extent to which the results can be explained by the models. “*-*” suggests that at *P* = 0.05 the relationship is not significant. Bold allometric slopes represent that they are significantly different from 1 at *P* = 0.05. Values in the same row sharing the same letter(s) are not significantly different at *P* = 0.05.

## Discussion

### Reproductive allocation

Our results showed that spike biomass, reproductive tiller biomass and SPIKE / TILLER biomass ratio were significantly larger, but seed setting rate which was smaller in the marginal than in the central zone of clonal patches. This suggests that reproductive allocation of *L*. *chinensis* ramets was dependent on their positions within the large clonal patches. Compared with tillers growing in the central zone of patches, tillers in marginal zones faced less severe intraclonal competition, thus showing a greater tiller size and spike biomass. But the biomass of culms and leaves did not change (unpublished data). That is, the biomass of spikes and tillers did not increase at the cost of the biomass of culms and leaves. What are the mechanisms underlying larger tillers and greater spike biomasses in the marginal than in the central zones? First, the resources available to tillers were most likely in shortage in the central zones due to resource depletion of previous tillers; meanwhile, soil nutrients were left relatively more available in marginal zones. Secondly, probably pathogens or allelopathetic substances may have accumulated in the central zones [55]. Finally, tillers in the marginal zones can take up most of the surrounding resources and reduce nutrient supply to tillers in the central zones [56]. Despite larger SPIKE / TILLER biomass ratio in marginal than in central zones, the seed setting rate was smaller in marginal than in central zones. What is underlying this paradox? It may be that the density of *L*. *chinensis* tillers was lower in marginal than in the other zones, and therefore the chances of fertilization were lower in marginal than in central zones [57]. However, for the small clonal patches, the area was too small to have an obvious effect on reproductive allocation in central or marginal zones. It meant that the position of patch had no influence on reproductive allocation of *L*. *chinensis* in small patches.

Our results also indicated that clonal patch size had a negative effect on SPIKE / TILLER biomass ratio, and a positive effect on SEED / SPIKE biomass ratio. Reproductive tillers in larger clonal patches would experience more intensive intraclonal competition for nutrients; as a result, they would allocate more resources to vegetative growth at the expense of sexual reproduction [58], and to maintain their survival and competitive ability [59]. However, reproductive tiller biomass did not change with clonal patch size despite the increase of intraclonal competition. In spite of this, spike allocation to seeds was as great as possible, probably at the expense of spike stalk [26]. Therefore, *L*. *chinensis* increased the efficiency of sexual reproduction through the increase of seed setting when clonal patches became larger.

### Size-dependent effects on reproductive allocation

The significant positive relationships between spike and reproductive tiller biomasses, with slopes significantly greater than 1, suggested that the allocation to spikes was an allometric process, which is largely dependent on tiller size. The spike could get proportionally more biomass allocation than the vegetative organs. This meant that a reproductive tiller would maximize its reproductive output [7]. The relationships between mature seed and spike biomasses followed significant allometric relationships with slopes significantly larger than 1. This indicated that spike-level reproductive allocation depended on spike size, and proportionally more biomass could be allocated to mature seeds within a spike. It is common that bigger spikes have more seed production, as to ensure high reproductive efficiency. In addition, the slopes of the regressions of spike vs. reproductive tiller biomasses, and of mature seed vs. spike biomasses were significantly different among the five patch size categories. This implied that size-independent effects also played a major role in spike and seed biomass allocation at the tiller and spike levels respectively [7].

More interestingly, the mature seed biomass vs. reproductive tiller biomass relationship shifted from non-allometric at the ≤ 5m^2^, 5–15m^2^ and 15–40m^2^ patch size categories to significant allometric at the 40–100m^2^ and ≥ 100m^2^ patch size categories. It meant that the driver of SEED / TILLER biomass ratio variation changed from size-independent to size-dependent effects. The non-allometric reproductive strategies at the ≤ 5m^2^, 5–15m^2^ and 15–40m^2^ patch size categories suggested that the seed allocation at tiller level was independent of tiller size on relatively small clonal patch. This might be because the seed is not only a reproductive, but also a storage organ. In other words, the size-independent effect also played an important role in regulating SEED / TILLER biomass ratio in *L*. *chinensis*. By contrast, such relationships followed significant allometric relationships at the 40–100m^2^ and ≥ 100m^2^ patch size categories, implying that the size-dependent effects played a primary role in relatively large clonal patches. In conclusion, size-dependent effects played an important role in the allocation of biomass to spikes at the tiller level, and to seeds at the spike level, during clonal expansion. However, these effects were only important for biomass allocation to seeds at the tiller level, when clones expanded to large clonal patches.

In conclusion, *L*. *chinensis* would modify its sexual reproductive allocation including the spike biomass allocation to a tiller scale and mature seed biomass allocation to a spike scale during clonal patch expansion. The allocation of resources to reproduction of the clonal ramets was size-dependent, and was also subjected to clonal size and ramet position within the clonal patch. These findings are important for a better understanding of the clonal expansion of *L*. *chinensis*, and will be helpful for developing appropriate management practices for the *L*. *chinensis*-dominanted grasslands.

## Supporting Information

S1 File Dataset on reproductive allocation characteristics of *Leymus*. *chinensis* clones at five patch size categories. (XLS)
